# Antifungal activities of Equol against *Candida albicans in vitro* and *in vivo*

**DOI:** 10.1080/21505594.2024.2404256

**Published:** 2024-09-12

**Authors:** Fen Wang, Jinping Zhang, Qian Zhang, Zhangyong Song, Caiyan Xin

**Affiliations:** a Nanobiosensing and Microfluidic Point-of-Care Testing Key Laboratory of LuZhou, The Affiliated Traditional Chinese Medicine Hospital of Southwest Medical University, Luzhou, Sichuan Province, China; b School of Basic Medical Science, Southwest Medical University, Luzhou, People’s Republic of China; c Department of blood transfusion, Zhejiang people’s hospital, Yichang, China; d Technical Platform for the Molecular Biology, Research Core Facility, Southwest Medical University, Luzhou, People’s Republic of China; e Southwest Medical University, Hemodynamics and Medical Engineering Combination Key Laboratory of Luzhou, Luzhou, People’s Republic of China

**Keywords:** *Candida albicans*, biofilm, equol, hyphal morphogenesis, systemic candidiasis, Ras1-cAMP-PKA signal pathway

## Abstract

*Candida albicans* is an opportunistic fungal pathogen that can cause systemic infections in immunocompromised individuals. Morphological transition and biofilm formation are major virulence factors of *C. albicans*. Moreover, biofilm enhances resistance to antifungal agents. Therefore, it is urgent to identify new and effective compounds to target the biofilm of *C. albicans*. In the present study, the antifungal activities of equol against *C. albicans* were investigated. *In vitro*, the microdilution analysis and spot assay result showed that equol exhibited potent inhibitory activities against *C. albicans*. Further investigations confirmed that the antifungal effects of equol involved interference with the transition from yeast to hypha and biofilm formation of *C. albicans*. In addition, transcriptome sequencing and reverse transcription-quantitative PCR (qRT-PCR) analysis showed that equol significantly downregulated the expression of several genes in the Ras1-cAMP-PKA pathway related to hyphae and biofilm formation and significantly upregulated the expression of the negative transcriptional repressors *RFG1* and *TUP1*. Moreover, equol effectively reduced the production of cAMP, a key messenger in the Ras1-cAMP-PKA pathway, while supplementation with cAMP partly rescued the equol-induced defects in hyphal development. Furthermore, in a mouse model of systemic candidiasis (SC), equol treatment significantly decreased the fungal burden (liver, kidneys, and lung) in mice and local tissue damage, while enhancing the production of interleukin-10 (IL-10). Together, these findings confirm that equol is a potentially effective agent for treatment of SC.

## Introduction

*Candida albicans* is a common opportunistic fungus that is classified as a critically important pathogen by the guidelines of the World Health Organization [1]. *C*. *albicans* is known to colonize the oral cavity, vagina, and digestive tract of healthy individuals, and is considered pathogenic to those with compromised immune systems, particularly populations with acquired immunodeficiency syndrome, receiving chemotherapy, co-infected with other viral species, such as severe acute respiratory syndrome coronavirus 2, or receiving immunosuppressant therapy [2,3]. Moreover, *C. albicans* is reported to infect the superficial mucosa, dermis, and blood, with mortality rates of greater than 40% [4].

Biofilms are complex three-dimensional structures composed of a single species or mixed species microbial cells linked to host tissue or abiotic surfaces and imbedded in an extracellular polysaccharide substance, which provides protection to the microorganisms [5]. Biofilm is an important virulence factor of *C*. *albicans* that is largely responsible for resistance to antifungal agents and acts as physical barrier to host immune factors, thereby facilitating repeated infection [6]. Current clinical antifungal agents primarily include azoles, echinocandins, and polyenes. However, these agents have relatively low bioavailability, can cause severe side effects, and can promote the emergence of drug-resistant strains [7]. Thus, there is an urgent need for new antifungal agents against *C*. *albicans*.

Equol is a metabolite of daidzein produced by bacteria in the distal intestine and colon, and exhibits greater antioxidant and estrogenic activities than daidzein [8,9]. Previous studies have demonstrated that equol can protect against various cancers, diabetes, aging, osteoporosis, cardiovascular diseases, neurological diseases, and postmenopausal symptoms [10]. Moreover, equol is reported to exhibit antibacterial effects against *Clostridioides difficile* and carbapenem-resistant *Escherichia coli*, as well as antifungal effects against *C. albicans* [11–13]. However, the underlying mechanism of this anti-*Candida* effect and the application of equol in the murine model of systemic candidiasis (SC) have not been reported.

Therefore, the aims of the present study were to assess the *in vitro* and *in vivo* antifungal effects of equol. The results of *in vitro* studies suggest that the antifungal effects of equol may involve the Ras1-cAMP-PKA signaling pathway to inhibit the growth, hyphal morphogenesis, and biofilm formation of *C*. *albicans*. Moreover, *in vivo*, equol reduced the fungal burden in a murine model of SC. Taken together, these findings highlight the antifungal activities of equol as a promising antifungal agent for clinical application.

## Materials and methods

### Strain, culture media, and chemicals

*C*. *albicans* strain SC5314 (ATCC® MYA-2876™; American Type Culture Collection Manassas, VA, USA) was grown on yeast peptone dextrose (YPD) media (Solarbio, Beijing, China). Equol was purchased from Shanghai Yuanye Bio-Technology Co., Ltd., Shanghai, China. Amphotericin B (AmB) and Colorimetric 2,3-bis (2-methoxy-4-nitro5-sulfophenyl) − 2 H-tetrazolium-5-carboxanilide sodium salt (XTT) were purchased from Macklin Biochemical Co., Ltd., Shanghai, China.

### Broth microdilution assay

The minimum inhibitory concentration (MIC) of equol against *C*. *albicans* strain SC5314 was determined using the Clinical and Laboratory Standards Institute document M27-A4 (Reference Method for Broth Dilution Antifungal Susceptibility Testing of Yeasts) [14]. Briefly, the equol and AmB were dissolved in dimethyl sulfoxide (DMSO, Sigma, Shanghai, China) to prepare stock solution of 50 mg/mL and 1024 μg/mL, respectively. The tested concentrations of equol and AmB were 0.0625–2 mg/mL and 0.0313–16 μg/mL, respectively. To prepare cell suspensions, a single colony from YPD plates was re-suspended in YPD liquid medium and cultured to a concentration of 1 × 10^6^ cells/mL. The activated yeast cells were dispersed in Roswell Park Memorial Institute (RPMI) 1640 medium (HyClone, Chengdu, China) to concentrations of 5 × 10^3^ cells/mL. 100 μL suspensions were then dispensed into triplicate wells of sterile 96-well microtiter plates. After incubation at 37°C for 24 h, the optical density of each well was measured at 600 nm using a microtiter plate reader (BioTek Instruments, Inc., Winooski, VT, USA). The minimal inhibitory concentration was defined as the lowest concentration of equol that suppressed fungal growth by 90%.

Next, a spot assay was conducted to determine the inhibitory activity of equol against *C*. *albicans* cultured on solid medium. Briefly, the *C. albicans* cells suspension was prepared by adjusting the concentration to 1 × 10^6^ cells/mL YPD liquid medium which contained 0.25 mg/mL equol and 1% DMSO as a control, and then the mixture was cultured at 37°C and 180 rpm for 12 h. Finally, 3 μL of 10-fold serially diluted yeast cultures was spotted onto YPD plates. After incubation for 48 h at 37°C, the fungal cells were imaged with a digital camera equipped with a 60-mm macro-lens (Canon Inc., Tokyo, Japan) for morphological analysis.

### Time-kill assay

The time-kill assay was performed as described in a previous report, but with slight modifications [15]. An overnight culture of *C. albicans* was washed and diluted to 1 × 10^5^ cells/mL in YPD medium containing 0.25 mg/mL equol or AmB, and incubated at 37°C. A portion of the cell suspension was collected at 2 h, 4 h, 8 h, 12 h, and 24 h, respectively. Diluted with PBS, and spread onto YPD plates using 100 µL diluted suspension. After incubation of the plates at 37°C for 48 h, the number of colony-forming units (CFUs) was quantified.

### Live/Dead fluorescent staining

Briefly, 1 × 10^6^ cells/mL of *C. albicans* growth in YPD medium was exposed to 0.25 mg/mL equol for 4 h, 8 h, and 12 h at 37°C, shaking at 180 rpm. Next, cells were washed with saline solution twice and stained with NucGreen and EthD-III for 15 min in the dark at room temperature. After washing with saline, the fungal suspension was then collected and observed under a microscope (Leica Microsystems GmbH, Wetzlar, Germany).

### Effect of equol on hyphal morphogenesis and colony morphology

The effects of equol on the yeast-to-hyphal phase transition of *C. albicans* were assessed as described in a previous study [16]. Briefly, an overnight culture of *C. albicans* was diluted to 1 × 10^6^ cells/mL in spider medium containing 0.0625 mg/mL, 0.125 mg/mL, 0.25 mg/mL of equol or 1% DMSO, and incubated at 37°C for 4 h under aerobic conditions with constant shaking at 180 rpm. Afterward, the cells were stained with calcofluor white solution (Sigma, Shanghai, China) supplemented with 10 μL of 10% potassium hydroxide solution at room temperature for 5 min. Then, the yeast cells and hyphal forms were observed under ultraviolet light (425 nm) with a fluorescence microscope (Leica Microsystems GmbH, Wetzlar, Germany). Inhibition of yeast-to-hyphal transition was quantified by counting the number of individual budded cells versus the number of hyphae in the population. More than 100 cells were counted for each sample in triplicate.

The effect of equol on the filamentous morphology of *C. albicans* was determined by culturing on spider solid medium. Briefly, 3 µL of activated yeast cells was spotted on the center of spider agar containing 0.25 mg/mL of equol. Solid agar without equol served as a control. After incubation at 37°C for 48 h, images of the filamentous morphology were captured using a digital camera.

### Effect of equol on biofilm formation by C. albicans and preformed biofilms

The metabolic activity of biofilm was quantified with the colorimetric XTT reduction assay [17]. Briefly, for the biofilm formation assay, *C. albicans* suspension was prepared in RPMI 1640 medium at a concentration of 1 × 10^6^ cells/mL with the 0.25 mg/mL concentration of equol, and added 200 μL to the wells of sterile 96-well plates (Becton Dickinson, Beijing, China). As a control, 200 μL of RPMI 1640 medium containing 1% DMSO without equol was added to selected wells. Then, the plates were incubated at 37°C for 24 h.

For the preformed biofilms, 200 μL of activated yeast cells in suspension (1 × 10^6^ cells/mL) was added to the wells of a 96-well plate. After incubation at 37°C for 24 h, the plate was washed two times with PBS. Then, 200 μL of RPMI 1640 medium containing 0.25 mg/mL of equol was added to the wells. As a control, 200 μL of RPMI 1640 medium containing 1% DMSO without equol was added to selected wells of the plate. After incubation at 37°C for 24 h, the metabolic activity of the biofilm was determined with the XTT reduction assay, as described above.

Furthermore, the effects of equol on biofilm formation by *C. albicans* and preformed biofilm was qualified with a confocal laser scanning microscope (CLSM; Leica Microsystems GmbH). Biofilms were formed on glass cover slips coated with poly-L-lysine in the wells of 12-well cell culture plates, as described above. After incubation, the cover slips were transferred to the wells of new 12-well plates and washed twice with sterile PBS. After staining with calcofluor white, the formed biofilm was observed with a CLSM.

### Transcriptome and quantitative reverse transcription polymerase chain reaction (qRT-PCR) analyses

*C. albicans* cells (1 × 10^6^/mL) were cultured in YPD liquid medium with or without equol (0.25 mg/mL) at 37°C for 24 h as described above and then harvested. Total RNA was extracted using the RNAiso Plus kit (TaKaRa Biotechnology Co., Ltd., Dalian, China) in accordance with the manufacturer’s instructions. The transcriptome data were processed by Biomarker Technologies (Qingdao, China) using a commercial sequencing platform (Oxford Nanopore Technologies, Oxford, England). Raw sequence data were deposited in the Genome Sequence Archive of the Beijing Institute of Genomics (accession no. CRA011429). The criteria for identification of differentially expressed genes (DEGs) were |fold change| ≥2 and false-discovery rate ≤0.01.

Total RNA was extracted from *C. albicans* cells cultured in YPD containing 0.25 mg/mL of equol at 37°C for 24 h and amplified by RT-qPCR using the PrimeScript™ RT reagent kit with gDNA Eraser (TaKaRa Biotechnology Co., Ltd., Dalian, China) with TB Green® Premix Ex *Taq*™ II polymerase (TaKaRa Biotechnology Co., Ltd., Dalian, China) and the primers listed in Table S1. Relative expression of the target genes was determined using the 2^−^△△^CT^ method against β-actin as an internal control [18].

### Intracellular content of cyclic adenosine monophosphate (cAMP) and cAMP rescue experiments

The effect of equol on the intracellular concentration of cAMP was determined as previously described [19]. In brief, *C. albicans* cells (1 × 10^6^/mL) were cultured in RPMI 1640 medium containing 0.25 mg/mL of equol at 37°C for 2, 4, and 24 h. *C. albicans* cells (1 × 10^6^/mL) in RPMI 1640 medium without equol served as a control. Then collected by centrifugation at 3000 × *g* for 10 min, washed 2 times with sterile water, weighed the dry weight, frozen in liquid nitrogen and thawed at room temperature repeatedly, and finally suspended in sterile water with 5% trichloroacetic acid. Then breaking by ultrasonication, the supernatant was neutralized with water-saturated ether and then subjected to freeze-drying. The intracellular content of cAMP was measured using a commercial enzyme-linked immunosorbent assay kit (Abmart Medical Technology Co., Ltd., Shanghai, China) in accordance with the manufacturer’s protocol.

N6-2”-O-Dibutyryladenosine-3,”5’-cAMP (dbcAMP) (Aladdin Bio-Chen Technology Co., Ltd., Shanghai, China) was prepared as a 100 mM stock solution in water. Activated *C. albicans* cells were diluted to 1 × 10^6^/mL in RPMI 1640 medium containing 0.25 mg/mL equol and 15 mM db-cAMP in a sterile tube. Untreated cells served as a control. After incubation at 37°C for 4 h, cell morphology was assessed as described above.

### Antifungal activity of equol in vivo

All experimental protocols were approved by the Southwest Medical University Institutional Animal Care and Use Committee (2020540). Male BALB/c mice (age, 6–8 weeks; body weight [BW], 20–25 g) were purchased from Chongqing Tengxin Biotechnology Co., Ltd. (Chongqing, China) and housed in an animal care facility with *ad libitum* access to food and water. Immunosuppression was induced in the mice by intravenous injection of cyclophosphamide at 200 mg/kg BW for 3 days. Then, the mice were infected *C*. *albicans* cells (100 μL at 1 × 10^7^ cells/mL) by injection into the tail vein. The SC mice received equol at 20 mg/kg BW/day via oral-gastric gavage at 2 h after successful infection and then daily for 7 days. The same volume of normal saline and AmB (7.5 mg/kg BW/day) were used in the control group. All animals were euthanized by cervical dislocation after anesthesia with pentobarbital (50 mg/kg).

The fungal load was measured along with histopathological analysis of liver, lung, kidney, and serum samples to evaluate the efficacy of equol against *C. albicans* infection at days 1, 4, and 7. Portions of the collected liver, lung, and kidney samples were used to prepare homogenates, which were diluted and plated on YPD agar. The number of colony-forming units per gram of tissue was determined after incubation at 37°C for 24 h. The remaining tissues were fixed with 10% methanol, embedded in paraffin, and cut into thin sections, which were stained with periodic acid-Schiff (PAS) stain (Beijing Solarbio Science & Technology Co., Ltd., Beijing, China) for microscopic observations.

Serum levels of interleukin (IL)-2, IL-4, IL-6, IL-10, IL-17A, interferon (IFN)-γ, and tumor necrosis factor (TNF-α) on days 1, 4, and 7 were measured with a BD™ Cytometric Bead Array Mouse Inflammation Kit (BD Biosciences, San Jose, CA, USA) in accordance with the manufacturer’s protocol. Then, the samples were subjected to flow cytometry with a BD FACSCalibur Flow Cytometer (BD Bioscience). The results were analyzed using FCAP Array Software Version 3.0 (BD Bioscience).

The above experimental methods followed the ARRIVE guidelines. https://arriveguidelines.org/sites/arrive/files/documents/Author%20Checklist%20-%20Full.pdf.

### Statistical analysis

Each experiment was repeated three times. All statistical analyses were performed using GraphPad Prism software version 8.0 (GraphPad Software Inc., La Jolla, CA, USA). One-way analysis was performed for comparisons among the groups. Cytokine levels were compared with the unpaired two-tailed Mann – Whitney (nonparametric) test. The results are presented as the mean of three independent experiments ± standard deviation (SD). A probability (*p*) value of <0.05 was considered statistically significant.

## Results

### In vitro antifungal activities of equol against the growth and hyphal morphogenesis of C. albicans

**Figure 1.**
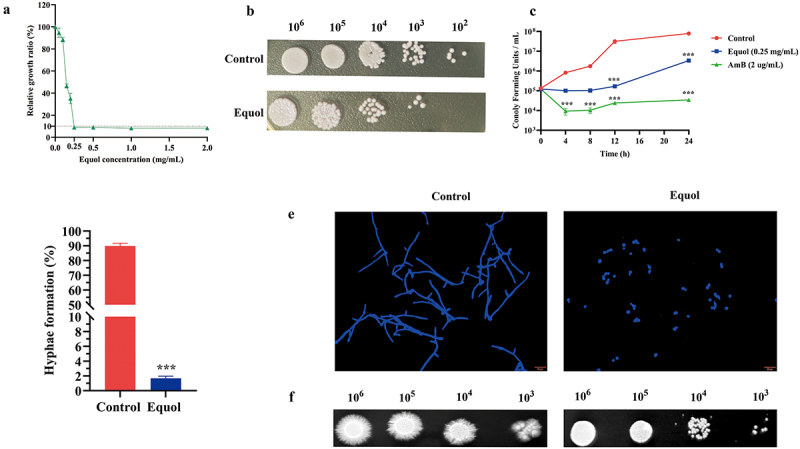
The inhibitory effects of equol on growth and hyphal morphogenesis of *C. albicans*. (a) Relative growth curve of *C. albicans* treated with various concentrations of equol. (b) Effect of equol on *C. albicans* growth. Control group was treated with 1% DMSO. Equol group was treated with equol (0.25 mg/mL). After overnight culture, *C. albicans* cells were spotted on YPD agar plates and cultured at 37°C for 48 h. The concentrations of *C. albicans* cells were 10^2^, 10^3^, 10^4^, 10^5^, and 10^6^. (c) Time-kill curves of *C. albicans* treated with equol at 0.25 mg/mL, and AmB at 2 μg/mL. (d) Inhibitory effects of equol against hyphal formation of *C*. *albicans*. (e) Effect of equol on *C. albicans* hyphal formation in liquid spider medium at 37°C for 4 h. Images of cellular morphology were obtained using a fluorescence microscope. Scale bar = 20 µm. (f) Effect of equol on *C. albicans* hyphal formation on solid spider medium. Plates were incubated at 37°C for 48 h. The concentrations of *C. albicans* cells were 10^3^, 10^4^, 10^5^, and 10^6^. Images of the filamentous morphology were captured with a digital camera. ***p < 0.001 vs. The control group.

To evaluate the antifungal potential of equol against *C. albicans*, we determined the MIC. The MIC of equol against the *C. albicans* was 0.25 mg/mL (Figure 1a). For AmB, the MIC for the *C. albicans* was 2 μg/mL. The results of the spot assay showed that the growth of *C. albicans* was inhibited by equol solid medium (Figure 1b). Time-kill curves revealed that equol effectively inhibited the growth of *C. albicans* within 24 h of incubation compared with control group (Figure 1c). In addition, a live/dead fluorescent staining assay was performed. In the control groups (4 h, 8 h, and 12 h), numerous green cells were visualized, indicating most of the cells had intact membrane integrity. Whereas, the number of dead cells stained red were increased gradually by increasing the treatment time with equol (Supplementary Fig S1). These results confirmed that equol possessed robust antifungal activities against *C. albicans*.

Moreover, the morphological effects of equol (0.25 mg/mL) on growing hyphae were also examined. *C. albicans* cells treated with equol (0.25 mg/mL) completely lacked hyphae and appeared only in the yeast form with a significantly lower ratio of hyphal cells as compared to the control group (1.68% ± 0.28%, *p* < 0.001) (Figure 1d). Similarly, compared with control group, *C*. *albicans* cultured in Spider liquid medium supplemented with 1/4 × MIC or 1/2 × MIC equol had comparatively shorter hypha (Supplementary Fig. S2). In the hyphae-inducing liquid Spider medium containing 1 × MIC (0.25 mg/mL) of equol, nearly all *C. albicans* cells grew in the yeast form (Figure 1e). Additionally, *C. albicans* cells cultured on solid Spider medium supplemented with equol were devoid of filamentous growth (Figure 1f). Collectively, these results showed that equol suppressed filament development and inhibited yeast-to-hyphae transition of *C. albicans.*

### Equol inhibited biofilm formation and eradicated preformed biofilm

**Figure 2.**
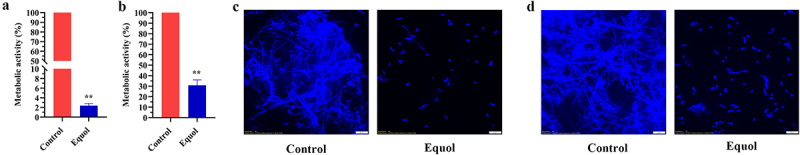
Effect of equol on *C. albicans* biofilm formation and preformed biofilms. Effect of equol on biofilm formation (a) and preformed biofilms (b) metabolic activity of *C. albicans* by the XTT reduction assay. The results are presented as the average of three independent experiments ± SD. Morphological changes of *C. albicans* biofilm formation (c) and preformed biofilms (d) by confocal laser scanning microscopy. **p < 0.01 vs. The control group.

The antibiofilm activities of equol were evaluated using the XTT reduction assay and viability was expressed as a percentage of metabolic activity. Equol (0.25 mg/mL) significantly decreased the formation and metabolic activity of the biofilm (Figure 2a) (*p* < 0.01). Pretreatment with equol (0.25 mg/mL) decreased biofilm formation by 70% (Figure 2b). In addition, the antibiofilm potential of equol was further observed with a CLSM. Images obtained with a CLSM showed that the hyphae of the control group were comparatively denser and more compact (Figure 2c,d). Meanwhile, biofilms formed by *C*. *albicans* treated with equol (0.25 mg/ml) showed minimal hyphal organization and consisted mostly of yeast cells.

### Equol induced changes to the C. albicans transcriptome

**Figure 3.**
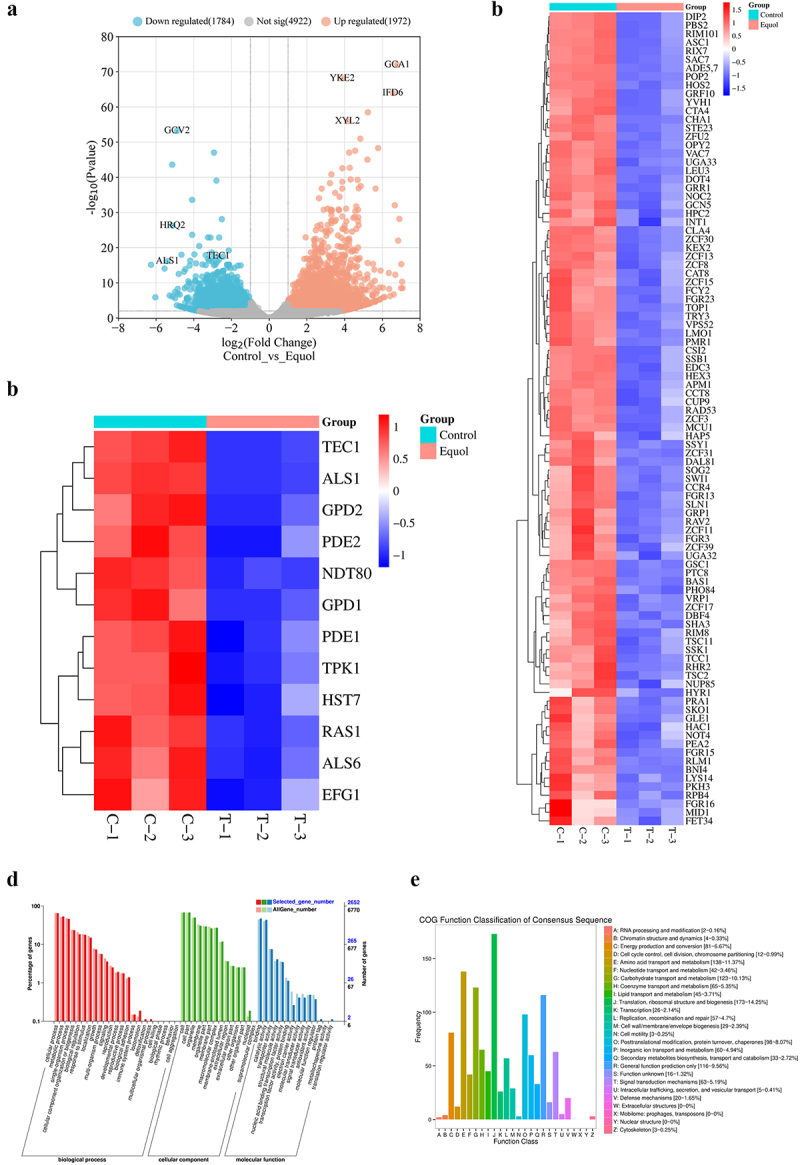
Transcriptome analysis after treatment with equol. (a) Volcano plot of DEGs. The cutoff values fold change > 2 and FDR < 0.01 were utilized to identify DEGs. The sapphire blue dots indicate downregulated genes (1784) and the orange dots indicate upregulated genes (1972). Heatmap plot of filamentous growth and biofilm formation genes (b) and Ras1-cAMP-pka pathway (c) in *C. albicans* after equol treatment. (d) GO terms of DEGs. The horizontal axis is the functional category and the vertical axis is the annotated with the percentage of the total number of genes (left). (e) Clusters of orthologous groups (COG) functional enrichment of DEGs.

The results of this study demonstrated that equol effectively inhibited hyphal and biofilm formation by *C. albicans*. To further investigate the underlying mechanisms, equol-induced changes to the *C. albicans* transcriptome were identified by RNA sequencing with the Illumina platform (Illumina, Inc., San Diego, CA, USA). As shown in Figure 3a, after treatment by equol, 3756 significantly DEGs (1972 upregulated and 1784 downregulated) were identified by comparison of the control and equol-treatment groups. Because equol decreased filamentous growth and biofilm formation, we focused on genes related to hyphae. Among 1784 downregulated genes, 149 genes are related to filamentous growth and biofilm formation (Figure 3b) and Ras1-cAMP-PKA pathway (Figure 3c) in *C. albicans* after equol treatment.

According to the transcriptome sequencing results, treatment with equol significantly altered the expression levels of genes involved in hyphal and biofilm formation by *C*. *albicans*. As shown in Table S2, equol downregulated expression of biofilm-related genes, transcriptional regulators of filamentous growth, and mitogen-activated protein kinase (*ALS1*, *ALS6*, *EFG1*, *NDT80*, *HST7*, *TEC1* and *PRA1*). Accordingly, *ALS1* and *ALS6*, contribute to the adhesion and aggregation of yeast cells and are essential to biofilm formation [20]. *EFG1* is a major transcription factor that acts as a morphological regulator and involved in promoting filamentous growth and regulation of the expression of several genes associated with invasion and/or biofilm formation [21]. *NDT80* is required for hyphal growth in response to different filament-inducing cues and for regulation of the expression of genes related to hyphal growth and those characterizing the filamentous transcriptional program [22]. *HST7*, which encodes a serine/threonine-protein kinase STE7 homolog protein, plays a crucial role in mating [23]. Moreover, *TEC1* and *PRA1*, the master regulatory genes involved in biofilm formation, play crucial roles in hyphal development [24,25]. Notably, *TEC1* is important for regulating the morphological switch between yeast and hypha [26]. Meanwhile, equol treatment also downregulated the expression of genes related to the Ras1-cAMP-PKA signaling pathway (*RAS1*, *PDE1*, *PED2*, and *TPK1*) [27]. In addition, *RFG1* and *TUP1*, negative regulators of hyphal and filamentation-related genes, were upregulated, thereby further substantiating the anti-hyphal potential of equol [28,29].

### Equol affects expression of genes related to biofilm formation and cAMP production

**Figure 4.**
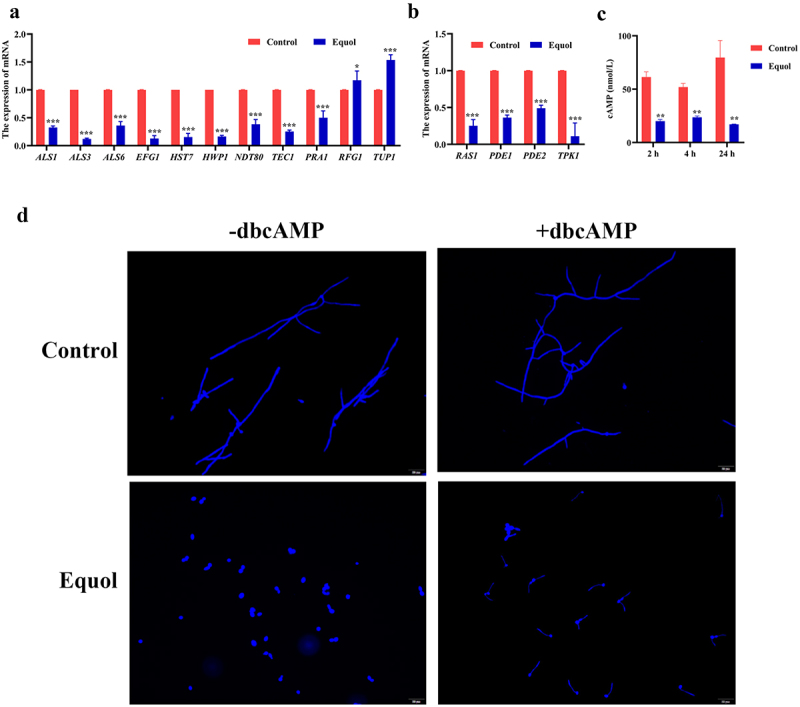
Expression analysis of genes associated hyphae production, biofilm formation, and the Ras1-cAMP-pka signaling pathway of *C. albicans* after equol treatment. (a) DEGs involved in hyphal development and biofilm formation. (b) DEGs involved in Ras1-cAMP-pka signal pathway. (c) equol reduced intracellular cAMP levels. (d) Defective morphological transition of *C. albicans* caused by equol was reverted by the addition of exogenous db-cAMP. Data are presented as the mean ± SD. *p < 0.05, **p < 0.01, and ***p < 0.001 vs. The control group.

The effects of equol on genes related to hyphal growth, biofilm formation, and the Ras1-cAMP-PKA signaling pathway were identified by RNA-seq and confirmed by qRT-PCR analysis. The results showed that equol treatment downregulated expression of *ALS1*, *ALS3*, *ALS6*, *EFG1*, *HST7*, *HWP1*, *NDT80*, *TEC1*, and *PRA1*, as well as genes related to the Ras1-cAMP-PKA signaling pathway (*RAS1*, *PDE1*, *PDE2*, and *TPK1*), while upregulating expression of *RFG1* and *TUP1* (Figure 4a,b). These data suggest that equol inhibits hyphal transformation and biofilm formation of *C. albicans*.

To further explore the effects of equol on the Ras1-cAMP-PKA signaling pathway, cAMP production by *C. albicans* cells was determined at 4, 12, and 24 h after treatment with equol. The results showed that equol significantly reduced production of cAMP at all time points (Figure 4c). Moreover, the addition of exogenous cAMP restored hyphal formation in the equol-treated groups (Figure 4d). Together, these data suggested that Ras1-cAMP-PKA signaling pathway may play an important role in equol inhibiting filamentous growth of *C. albicans*.

### Therapeutic effects of equol on the SC murine model

**Figure 5.**
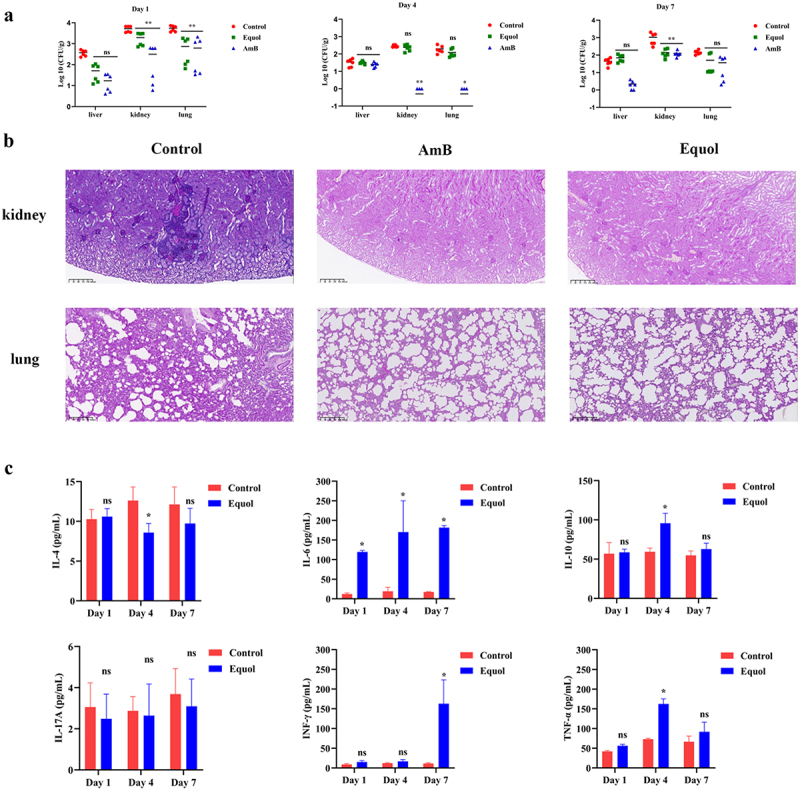
Therapeutic effect of equol on SC in mice. (a) Fungal burdens of the liver, kidney, and lung tissues. The SC model mice were treated with equol by gastric gavage at 20 mg/kg BW/day, and the fungal burden was measured on posttreatment days 1, 4, and 7. (b) Histological analysis of mice. pas-stained sections were prepared from the kidneys and lungs of mice on day 1 after treatment with equol at 20 mg/kg BW/day. Scale bar = 100 μm. (c) Serum cytokine concentrations. Control group: mice injected with *C. albicans* suspended in normal saline and daily treatment with DMSO diluted in normal saline. Equol group: mice injected with *C. albicans* suspended in normal saline and daily treatment with 20 mg/kg of equol. AmB: mice injected with *C. albicans* suspended in normal saline and daily treatment with 7.5 mg/kg of AmB. CFU: colony forming units. ns: *p* > 0.05, *p < 0.05, **p < 0.01 vs. The control group (analysis of variance or unpaired *t*-test).

The fungal burden was measured and histopathological analysis of SC mouse tissues was conducted to assess the effects of equol administration. As shown in Figure 5a, the fungal burden of lung tissues was decreased one day after treatment with equol (*p* < 0.05). Furthermore, the fungal burden of the kidney tissues was decreased on days 1 and 7 after treatment with equol (*p* < 0.05). However, treatment with equol had no significant effect on the fungal burden of the liver tissues from day 1 to 7 (Figure 5a).

The results of histopathological analysis with PAS stain were consistent with the fungal burden measurements (Figure 5b). Moreover, abnormalities of the kidney and lung tissues were analyzed by staining with PAS. As shown in Figure 5b, the histopathological examination revealed the presence of fungal cells in the control group compared with the equol treated group or AmB treated group. The results suggest that treatment with equol was efficacious in SC mice.

CD4^+^ T cells (Th1, Th2, and Th17) secrete cytokines in response to fungal infections [30]. IFN-γ and TNF-α are primarily secreted by Th1 cells, IL-4 by Th2 cells, and IL-17 by Th17 cells [31]. To determine the effects of equol on cellular immunity, mouse serum at 1, 4, and 7 days after *C. albicans* challenge and drug treatment was analyzed. As compared to serum from mice in the control group, equol treatment significantly increased levels of the pro-inflammatory cytokine IL-6 on days 1, 4, and 7 (Figure 5c). However, equol treatment had no significant effect on serum levels of the pro-inflammatory cytokine IL-17A. In addition, equol treatment significantly increased serum levels of the pro-inflammatory cytokines IFN-γ and TNF-α on day 7 and day 4, and the anti-inflammatory cytokine IL-10 on day 4 (Figure 5c). Moreover, serum levels of the anti-inflammatory cytokine IL-4 were significantly decreased on day 4 after equol treatment (Figure 5c).

## Discussion

*C. albicans* is the most common cause of invasive candidiasis worldwide, contributing a huge global health burden. The cost of treating candida infection was 1.4 billion annually in the US alone in 2019 [32–34]. At present, there are relatively few effective antifungal agents. Therefore, development of new antifungal agents is the main strategy. The hyphal morphology is a major virulence factor of *C. albicans* [35,36]. Yeast-to-hyphal transition plays a predominant role in the virulence of *C. albicans* and has been associated with invasion of the epithelial layer by breaching and damaging endothelial cells, while avoiding phagocytosis by macrophages and neutrophils, thigmotropism, and immune evasion by antigenic variation [36,37]. Moreover, hyphae, as the foundation of biofilm formation, not only enhance the invasive ability of *C. albicans*, but also limit the efficacy of antifungal agents [38]. Hence, inhibition of hyphal and biofilm formation is considered a viable strategy against *C. albicans* infection.

**Figure 6.**
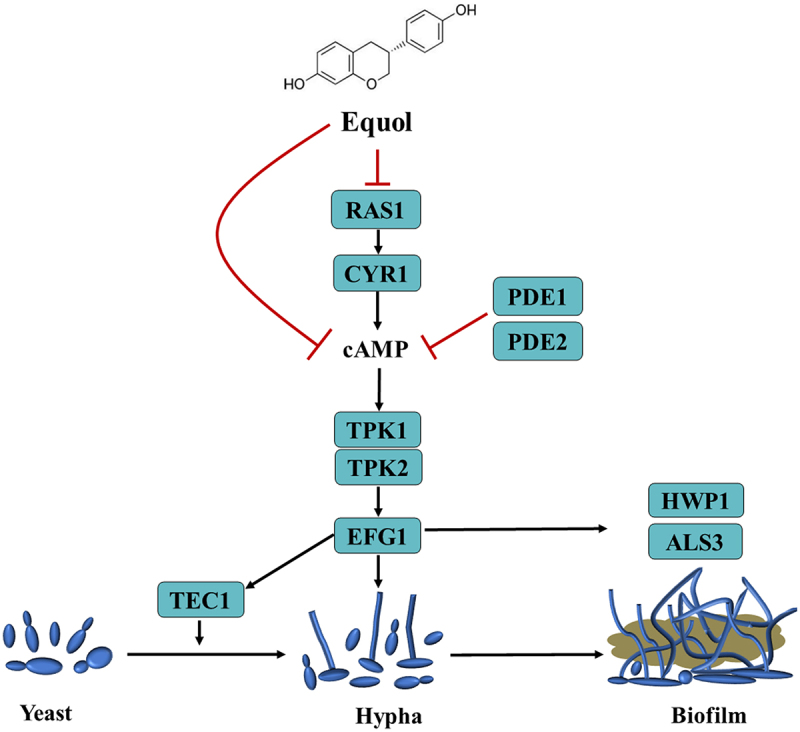
Mechanism of equol-induced inhibition of *C. albicans* biofilm formation by repression of the Ras1-cAMP-pka pathway. Equol significantly decreased the expression levels of genes associated with the Ras1-cAMP-pka pathway (*RAS1*, *PDE1*, *PDE2*, *TPK1*, and *EFG1*). In addition, equol reduced intracellular cAMP levels. However, exogenous db-cAMP restored hyphal formation in the equol-treatment group. Collectively, equol-mediated effects impeded activation of the Ras1-cAMP-pka signalling pathway and ultimately downregulated expression of *TEC1* and *EFG1*, thereby inhibiting hyphal growth and biofilm formation. *HWP1* and *ALS3*, which are involved in adherence, are also regulated by *EFG1*. Black arrows indicate activation and red lines indicate suppression.

In this study, the addition of equol in both liquid and solid spider medium significantly inhibited yeast-to-hyphal transition of *C*. *albicans* (Figure 1e,f). Yeast-to-hyphae transition is important for the development and maintenance of biofilm by *C*. *albicans* [39]. As compared to planktonic cells, mature biofilms are highly resistant (1,000-fold higher) to antifungal agents and provide protection against the host immune response [40]. Furthermore, various antifungal agents target biofilm formation. In the present study, equol was found to effectively inhibit biofilm formation by *C. albicans* and eradicate formed biofilms (Figure 2c,d). Similarly, the *in vivo* effects observed in the SC mice model demonstrated that equol had potent antifungal activity and can reduce the fungal burden (Figure 5a). The results of the current study showed that equol inhibited yeast-hyphal transition and biofilm formation of *C. albicans* may be used as an innovative treatment strategy. In addition, we showed that the antifungal and antibiofilm action of equol was associated with transcriptional control of gene expression, with equol treatment downregulating the expression of hypha-related genes (*ALS1*, *ALS3*, *ALS6*, *EFG1*, and *HWP1*) and biofilm regulators (*NDT80*, *TEC1*, and *PRA1*). In addition, *RFG1* and *TUP1*, negative regulators of hyphal and filamentation-related genes, were upregulated, thereby further substantiating the anti-hyphal potential of equol [28,29]. Meanwhile, equol treatment also downregulated the expression of genes related to the Ras1-cAMP-PKA signaling pathway (i.e. *RAS1*, *PDE1*, *PED2*, and *TPK1*), suggesting correlations of the genotypic and phenotypic results. The Ras1-cAMP-PKA signaling pathway is responsible for the adhesion, yeast-hyphal transition, biofilm formation, and virulence of *C. albicans* [41,42]. As a secondary messenger molecule, cAMP is crucial for activation of the Ras1-cAMP-PKA signaling pathway [43]. A previous investigation found that decreased intracellular levels of cAMP could block transition to the yeast form of *C. albicans* regardless of hypha-inducing conditions [44], implying that a certain level of cAMP is required for filamentation. Interestingly, exogenous cAMP rescued inhibition of hyphae transition (Figure 4d). Combined with the RNA-seq and qRT-PCR results, these data suggest that equol may inhibit the Ras1-cAMP-PKA signaling pathway, resulting in an alternation mechanism underlying the adherence, growth, yeast-to-hyphal transition, and biofilm formation of *C*. *albicans* (Figure 6). In addition, transcriptome results showed that DEGs were classified into 42 Gene Ontology (GO) categories (Figure 3d) and 23 clusters of orthologous groups (Figure 3e). And GO enrichment analysis revealed that the DEGs were also enriched in functions related to cellular process, growth, signaling, membrane, and catalytic activity.

Similarly, the *in vivo* effects observed in the SC mice model also demonstrated that equol had potent antifungal activity and can reduce the fungal burden (Figure 5a). The immune system protects the host from the harm of fungal pathogens through the stimulation of regulatory T cells to produce anti-inflammatory cytokines, such as IL-10 and IL-17A, which play important roles against SC [45]. IL-10 also plays an important role in inducing cytokine secretion by Th2 and Th17 cells [31]. In addition, during the infectious process, *C*. *albicans* increased production of the inflammatory cytokines INF-γ, TNF-α, IL-6, and IL-17. Moreover, IFN-γ, which is produced by Th1 cells, increases the fungicidal activity of macrophages and neutrophils in response to systemic or mucosal infections [29]. In this study, the anti-inflammatory responses of equol involved upregulation of the anti-inflammatory cytokines IL-10 and IL-4, and downregulation of the pro-inflammatory cytokine IL-17A. It has been shown that racemic equol inhibited the gene expression of several pro-inflammatory biomarkers such as IL-1A, IL-6, IL-8 and interleukin-1 receptor 2 and COX-1 [46,47]. However, the pro-inflammatory cytokine IL-6 was significantly up-regulated after treatment with equol, indicating other mechanisms underlying the ability of equol to influence production of IL-6. Hence, further studies are warranted to elucidate the underlying mechanisms.

In summary, the antifungal activities of equol against *C. albicans* were assessed both *in vivo* and *in vitro*. The results demonstrated that equol exhibited an inhibitory effect against yeast-to-hypha transformation and biofilm formation *in vitro*, and decreased the fungal burden in a mouse model of SC. Further investigations indicated that the mechanism of equol to inhibit biofilm formation may involve the Ras1-cAMP-PKA signaling pathway. Although gene knockout studies are needed to confirm the mechanism, the comprehensive assays conducted in this study revealed that equol has potential as a treatment to inhibit the proliferation of opportunistic fungal pathogens.

## Supplementary Material

Supplemental Material

## Data Availability

The raw sequence data of this study are openly available in Figshare at https://figshare.com/s/30797a3f690f64fc8173.
