# Supplementary material for: Antifungal activities of Equol against Candida albicans in vitro and in vivo
Source: Virulence. 2024 Sep 12;15(1):2404256. doi: 10.1080/21505594.2024.2404256 (PMC11409501; doi:10.1080/21505594.2024.2404256)
Supplement: Supplemental Material [file KVIR_A_2404256_SM1353.zip › supplementary table _clean copy.docx]

**Supplementary Table S1.** List of primers used for qRT-PCR experiments.

| **Genes** | **Protein annotation** | **Primer sequence (5’ - 3’)** |
| --- | --- | --- |
| *ACT1* | β-actin protein | Forward: CCAGGTATTGCTGAACGTATGC  Reverse: GAACCACCAATCCAGACAGAGT |
| *ALS1* | Als1p | Forward: GGATCTGTTACTGGTGGAGCTGTTG  Reverse: ATGTGTTGGTTGAAGGTGAGGATGAG |
| *ALS3* | Agglutinin-like protein | Forward: CCTATACCACTGCTACTACCGTT  Reverse: GTATGGTTGGTGTAATGAGGACG |
| *ALS6* | Als6p | Forward: AGTACGTCTGCCACCGATGCT  Reverse: AACTGCTTGCTGGGTCACTTGT |
| *EFG1* | Filamentous growth protein | Forward: AGTACCTATCCCACCACATGTATC  Reverse: GTTGTTACTCGTGGTCTGATTCC |
| *HST7* | Mitogen-activated protein kinase kinase | Forward: TCATCAGCTTCTTCTATAC  Reverse: TATTGAGGAAATGACAGTT |
| *HWP1* | Hyphal cell wall protein | Forward: TCTCTACGACTGAAGGTGCTATTC  Reverse: CCAATAATAGCAGCACCGAAAGTC |
| *NDT80* | Transcription factor NDT80 | Forward: AATCTACCCTGCAGTTCCTTCAG  Reverse: GTACTCTTGGTAGTAGTTTCCCCT |
| *PRA1* | Pra1p | Forward: AGGATAGCGACAGTGGCTCTGA  Reverse: GTGTGTGGCAATGCAGGTTAGC |
| *TEC1* | Conserved filamentation activator Tec1p | Forward: GTCCTATTTTCAACAGTCACGAGG  Reverse: CTTATTCTCTTTGTGGCTGGGAG |
| *RAS1* | Ras family GTPase | Forward: AAATCCGCTTTAACCAT  Reverse: AACCCTTCACCAGTTCTCA |
| *PDE1* | 3',5'-cyclic-nucleotide phosphodiesterase PDE1 | Forward: AGCAATCGTGTTGGAGTGTTCT  Reverse: TCGGCAATGGGTTCCTTGACA |
| *PDE2* | 3',5'-cyclic-nucleotide phosphodiesterase | Forward: CCATAATTTCCGCCACGCTGTG  Reverse: GCCCAATGCTGCAACCAATAAC |
| *TPK1*  *RFG1*  *TUP1* | Tpk1p  Rfg1p  chromatin-silencing transcriptional regulator | Forward: AGAAGTTCAAGATGTGACTTAT  Reverse: CATCATCAGAACCACCTTGT  Forward: AGTGGTGGTGGTGGTGGTAGT  Reverse: CACCTCCACCTCCACCTCCATT  Forward: CTTGGAGTTGGCCCATAGAA  Reverse: TGGTGCCACAATCTGTTGTT |

**Supplementary Table S2.** Changes to the expression levels of genes related to yeast-to-hypha transition, biofilm formation, and the Ras1-cAMP-PKA pathway in *C. albicans* after equol treatment.

| Gene identifier | Gene | Log_2_ fold change | *P* value | Significant | Regulation |
| --- | --- | --- | --- | --- | --- |
| CAALFM_C603700WA | *ALS1* | -5.397 | 6.89E-17 | Yes | Down |
| CAALFM_C306190CA | *ALS6* | -2.955 | 1.15E-05 | Yes | Down |
| CAALFM_C504910WA  CAALFM_CR03900WA  CAALFM_C200140WA  CAALFM_C304530CA  CAALFM_C106480CA  CAALFM_CR02140WA  CAALFM_C502290WA  CAALFM_C102840WA  CAALFM_C110220CA  CAALFM_CR02640WA  CAALFM_C100060WA | *EFG1*  *HST7*  *NDT80*  *TEC1*  *PRA1*  *RAS1*  *PDE1*  *PDE2*  *TPK1*  *RFG1*  *TUP1* | -1.791  -1.551  -2.243  -2.702  -2.807  -1.123  -2.338  -1.928  -2.203  1.397  1.778 | 0.00167  0.00150  5.55E-05  2.56E-18  0.00241  0.00020  0.0014  0.00017  1.49E-05  0.00286  1.26E-09 | Yes  Yes  Yes  Yes  Yes  Yes  Yes  Yes  Yes  Yes  Yes | Down  Down  Down  Down  Down  Down  Down  Down  Down  Up  Up |
