# Supplementary figures and images for: Antifungal activities of Equol against Candida albicans in vitro and in vivo
Source: Virulence. 2024 Sep 12;15(1):2404256. doi: 10.1080/21505594.2024.2404256 (PMC11409501; doi:10.1080/21505594.2024.2404256)

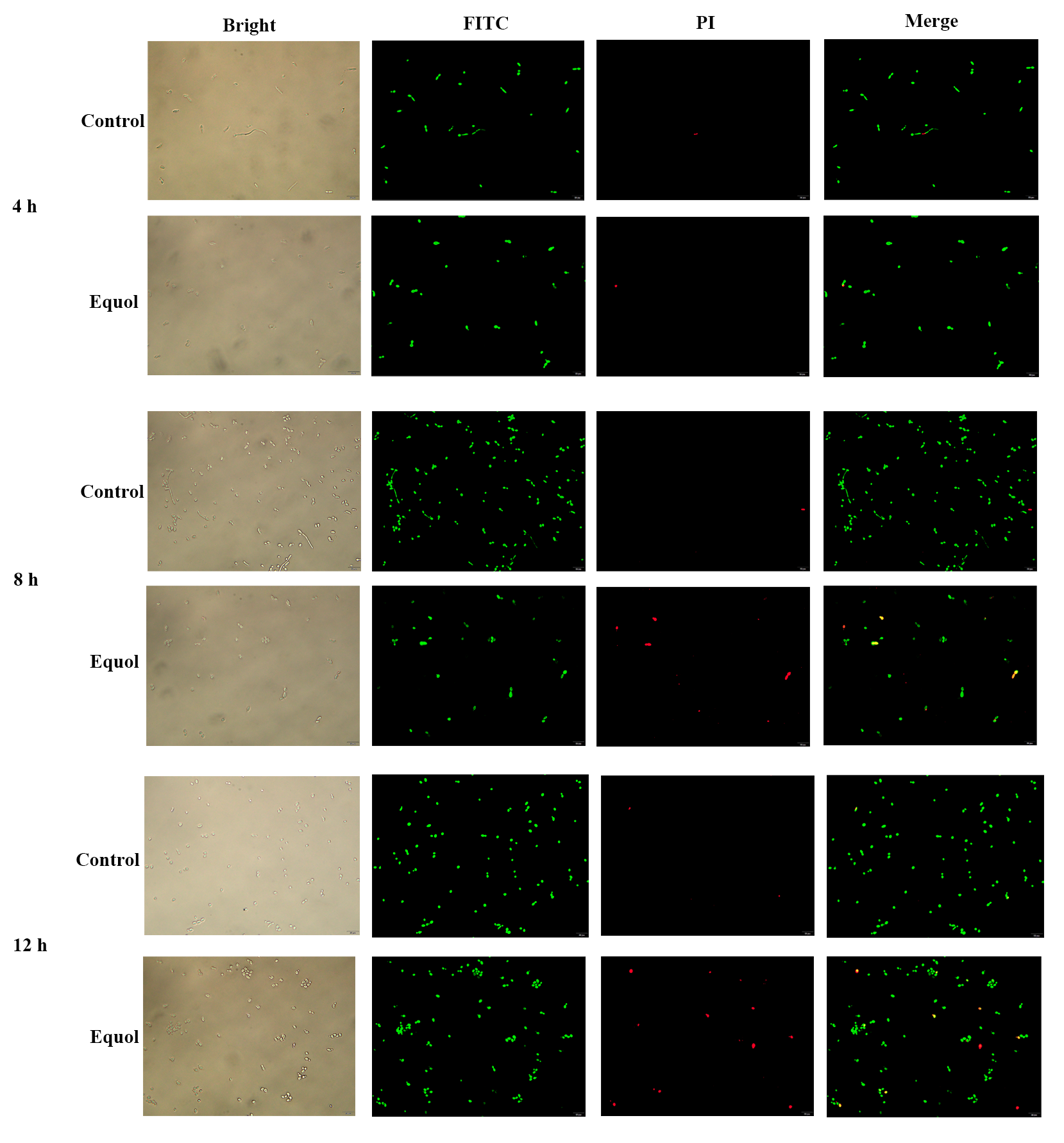

Supplement: Supplemental Material [file KVIR_A_2404256_SM1353.zip › Supplementary Fig 1.tif]

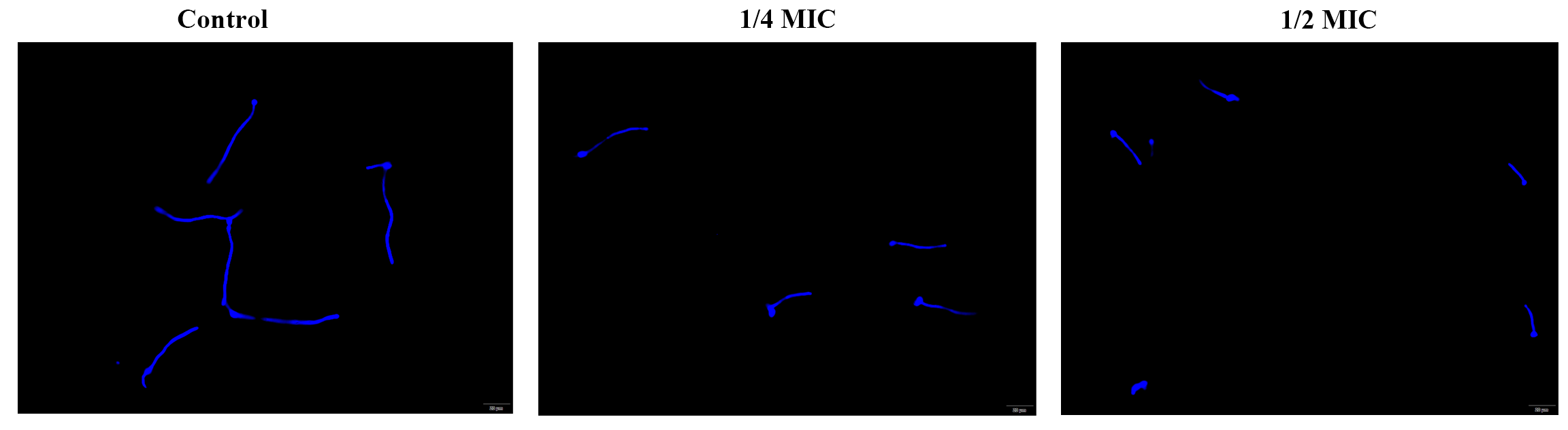

Supplement: Supplemental Material [file KVIR_A_2404256_SM1353.zip › Supplementary Fig 2.tif]
